# Triterpenoids CDDO and CDDO-EA Inhibit the Replication of Hepatitis B Virus by Modulating Nucleocapsid Assembly

**DOI:** 10.3390/ijms27010300

**Published:** 2025-12-27

**Authors:** Qiang Gao, Ge Yang, Ya Wang, Lu Yang, Jin Hu, Huiqiang Wang, Haiyan Yan, Kun Wang, Shuo Wu, Yuhuan Li, Jiandong Jiang

**Affiliations:** 1 CAMS Key Laboratory of Antiviral Drug Research, Beijing Key Laboratory of Technology and Application for Anti-Infective New Drugs Research and Development, NHC Key Laboratory of Biotechnology for Microbial Drugs, Institute of Medicinal Biotechnology, Chinese Academy of Medical Sciences and Peking Union Medical College, Beijing 100050, China; 2 State Key Laboratory of Bioactive Substances and Functions of Natural Medicines, Institute of Medicinal Biotechnology, Chinese Academy of Medical Sciences and Peking Union Medical College, Beijing 100050, China

**Keywords:** hepatitis B virus, triterpenoids, nucleocapsid assembly

## Abstract

Chronic hepatitis B virus (HBV) infection remains a global public health challenge, and the currently approved medications can not achieve a cure. Synthetic triterpenoids have shown promising therapeutic potential for liver pathologies. In our search for novel antiviral agents against HBV, we found that two triterpenoids, 2-cyano-3,12-dioxooleana-1,9-dien-28-oic acid (CDDO) and CDDO-ethyl amide (CDDO-EA), significantly inhibited HBV DNA replication. Further mechanistic investigation indicated that these two compounds did not significantly alter the levels of total HBV pgRNA, but dramatically reduced extracellular pgRNA and intracellular encapsidated pgRNA in a dose-dependent manner. Western blot analysis indicated minimal effects on core protein expression. Interestingly, using a particle gel assay, we observed that CDDO and CDDO-EA promoted the formation of empty capsids with no alteration in electrophoretic mobility. Moreover, we demonstrated that both compounds modulated the phosphorylation status of the core protein. Further cellular thermal shift assay (CETSA), surface plasmon resonance (SPR) assay, and molecular docking analyses collectively suggested that CDDO and CDDO-EA could bind directly to the dimer–dimer interfaces of HBV core protein. Finally, a synergistic effect was observed between CDDO-EA and lamivudine in reducing intracellular and extracellular HBV DNA levels. Our findings indicate that triterpenoids CDDO and CDDO-EA are new mechanistically type of HBV capsid assembly modulators and warranted for further development as lead compounds against HBV.

## 1. Introduction

Hepatitis B virus (HBV) is a small DNA virus belonging to the *Hepadnaviridae* family. HBV chronically infects an estimated 254 million people worldwide and approximately 15–40% of these carriers ultimately develop various severe liver diseases, including liver cirrhosis, hepatocellular carcinoma (HCC) and liver failure [1,2,3]. Current therapies of chronic hepatitis B include two formulations of alpha interferon (IFN-α) and five nucleos(t)ide analogues (NUCs) including lamivudine, telbivudine, adefovir, tenofovir and entecavir. Although these drugs can potently reduce the viral load and prevent liver disease progression, they fail to clear HBV infection reliably and eliminate the covalently closed circular (ccc) DNA. Moreover, long-term treatment with NUCs could cause virus drug-resistance. Therefore, the discovery and development of novel antiviral agents or treatment strategies with different mechanisms are needed [4,5,6,7].

Bardoxolone methyl (BARD or 2-cyano-3,12-dioxooleana-1,9-dien-28-oic acid-methyl ester (CDDO-Me)), a semi-synthetic triterpenoid, showed an improved effect in chronic kidney disease (CKD) associated with type 2 diabetes in a phase 2 clinical study [8,9,10] and recently was reported to suppress HBV and HCV replication in cell culture models [11]. Another synthetic triterpenoid CDDO-ethyl amide (CDDO-EA) was reported to ameliorate the progression of liver injury and fibrosis in a chronic carbon tetrachloride (CCl_4_)-induced mouse model of liver cirrhosis [12]. Moreover, CDDO-imidazole (CDDO-Im) can prevent ischemia-reperfusion (I/R)-induced liver injury in mice [13]. Together, these findings indicate that synthetic triterpenoids hold therapeutic potential for a range of liver pathologies.

In the present study, we evaluated the anti-HBV activities and mechanism of action of two triterpenoids, CDDO and CDDO-EA. Our results indicate that both compounds significantly reduced both intracellular and extracellular HBV DNA levels without affecting total pgRNA levels, yet potently diminished encapsidated pgRNA. We further demonstrated that both compounds uniquely induce the formation of empty capsids, with no alteration in electrophoretic mobility, through direct interaction with HBV core protein by binding to the dimer–dimer interfaces. Therefore, synthetic triterpenoids CDDO-EA and CDDO represent a chemically and mechanistically unique class of HBV capsid assembly modulators and warrant further development as novel antiviral agents against HBV infection.

## 2. Results

### 2.1. CDDO-EA and CDDO Inhibit HBV DNA Replication in Cell Cultures

**Figure 1:**
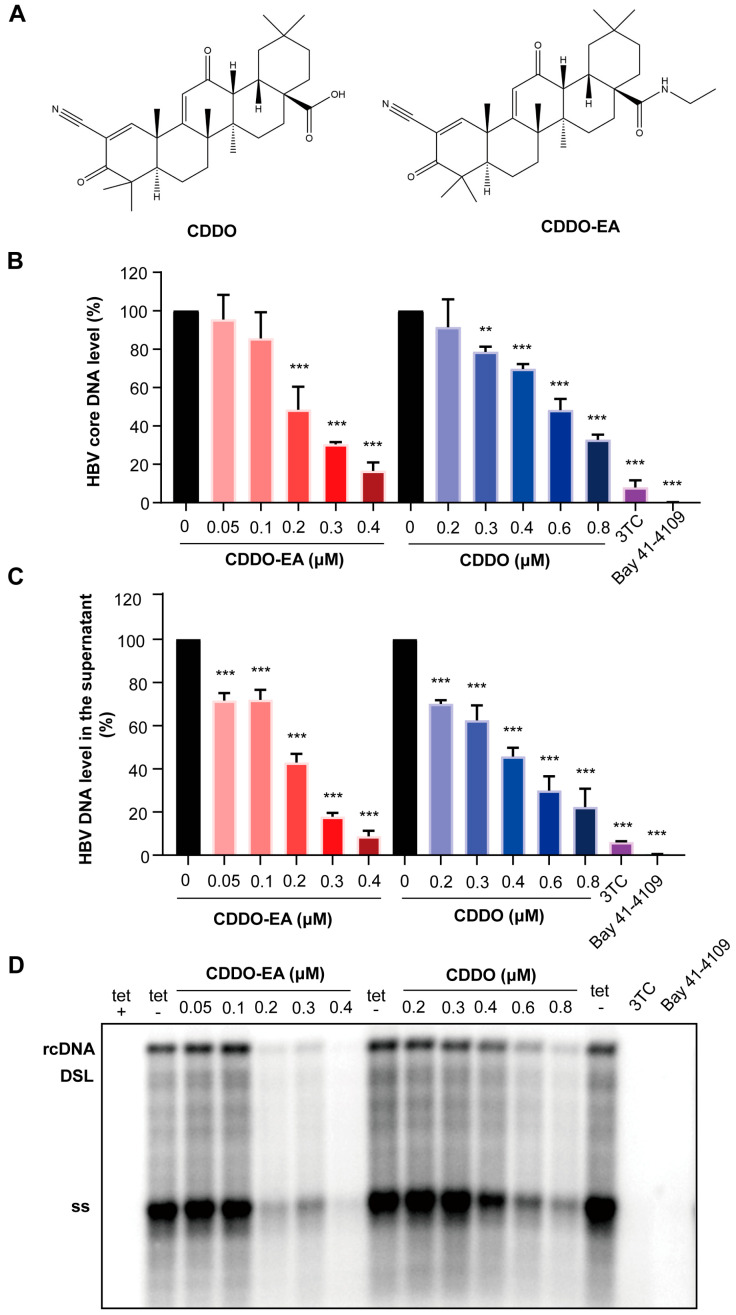
Two triterpenoids CDDO and CDDO-EA suppress the production of intracellular and extracellular HBV DNA. (**A**) Chemical structures of two compounds. (**B**–**D**) HepAD38 cells were cultured in the presence of tetracycline (tet +) or in the absence of tetracycline and mock-treated (0 or tet −) or treated with the indicated concentrations of the compounds CDDO-EA or CDDO for 6 days. Compounds 3TC (1 µM) and Bay 41-4109 (2 µM) were used as controls. (**B**,**C**) The intracellular and extracellular HBV DNA were quantified by a qPCR assay and expressed as the percentage of the mock-treated controls. The means and standard deviations (*n* = 3) were plotted. ** *p* < 0.01; *** *p* < 0.001. (**D**) HBV DNA replication intermediates were determined by Southern blot analysis. A DIG-labeled full-length minus-strand specific probe was used. rcDNA, relaxed circular DNA. DSL, double-stranded linear DNA. ss, single-strand, negative polarity DNA.

Taking advantage of HepAD38 that supports robust HBV replication in a tetracycline inducible manner [14], the antiviral activities of two synthetic triterpenoids CDDO and CDDO-EA (Figure 1A) was first examined in this cell line. To exclude the possibility that the observed antiviral effects were due to cytotoxicity, the cell viability was assessed using the PrestoBlue reagent. The results confirmed that the concentrations of CDDO (≤0.8 μM) and CDDO-EA (≤1 μM) used in the antiviral assays exhibited no significant cytotoxicity (Figure S1). The cells were also treated with HBV DNA polymerase inhibitor lamivudine (3TC) or core protein allosteric modulators (CpAMs), HAP derivative Bay 41-4109 [15] as positive controls. Both CDDO and CDDO-EA treatment led to a significant reduction in both intracellular and extracellular HBV DNA levels in a dose-dependent manner, as measured by qPCR (Figure 1B,C). Accordingly, Southern blot hybridization demonstrated a concomitant, dose-dependent suppression of all HBV DNA intermediates, including relaxed circular (rc) DNA, double-stranded linear (DSL) DNA and single-strand (ss) DNA (Figure 1D). We further examined their antiviral effects in another HBV-replicating cell line, HepG2.2.15, which demonstrated significant inhibitory effects on both intracellular and supernatant HBV DNA (Figure S2).

### 2.2. CDDO-EA and CDDO Inhibit HBV Nucleocapsid Assembly

**Figure 2:**
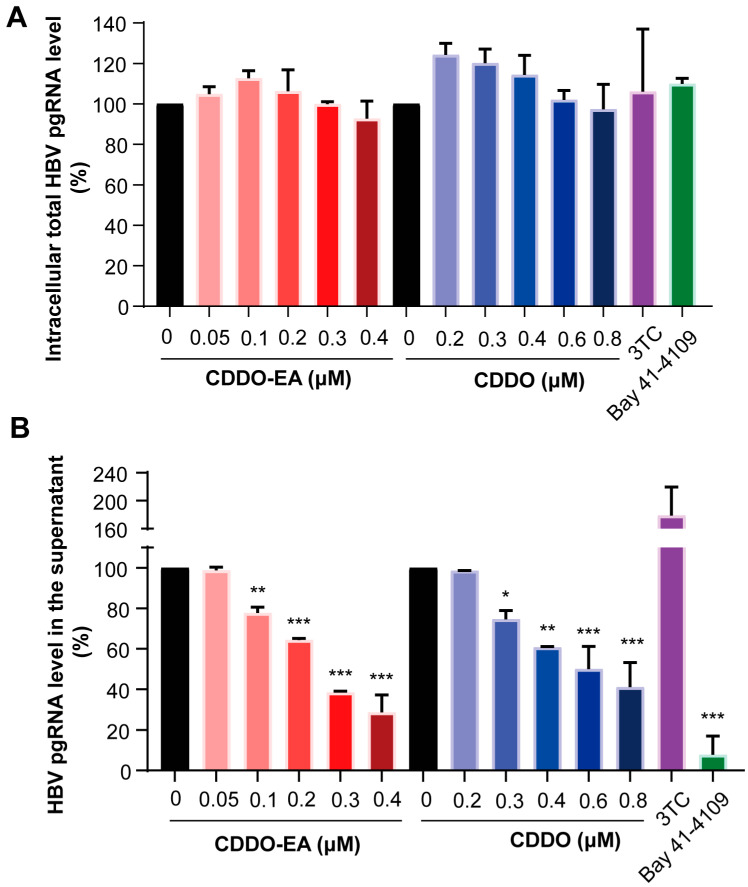
Effects of CDDO-EA and CDDO on total intracellular pgRNA and extracellular pgRNA levels. HepAD38 cells were cultured in the absence of tetracycline and mock-treated (0) or treated with the indicated concentrations of the compounds CDDO-EA and CDDO for 6 days. Intracellular total viral pgRNA (**A**) and pgRNA in the supernatant (**B**) were measured by qRT-PCR assay. Data are shown as mean ± SD from two independent experiments. Compounds 3TC (1 µM) and Bay 41-4109 (2 µM) were used as controls. * *p* < 0.05; ** *p* < 0.01; *** *p* < 0.001.

To investigate the underlying mechanism, total RNA in cells treated with the indicated compounds was then extracted and the level of pgRNA was analyzed by the RT-qPCR assay. As shown in Figure 2A, in agreement with their antiviral mechanisms and previous observations, neither 3TC nor Bay 41-4109 affected the intracellular levels of total HBV pgRNA. Similarly, CDDO-EA and CDDO also showed no significant effect on the pgRNA level (Figure 2A). Interestingly, subsequent evaluation of extracellular pgRNA levels revealed a significant reduction in cells treated with the two compounds (Figure 2B). In contrast, 3TC increased extracellular pgRNA levels, whereas Bay 41-4109 decreased them (Figure 2B).

**Figure 3:**
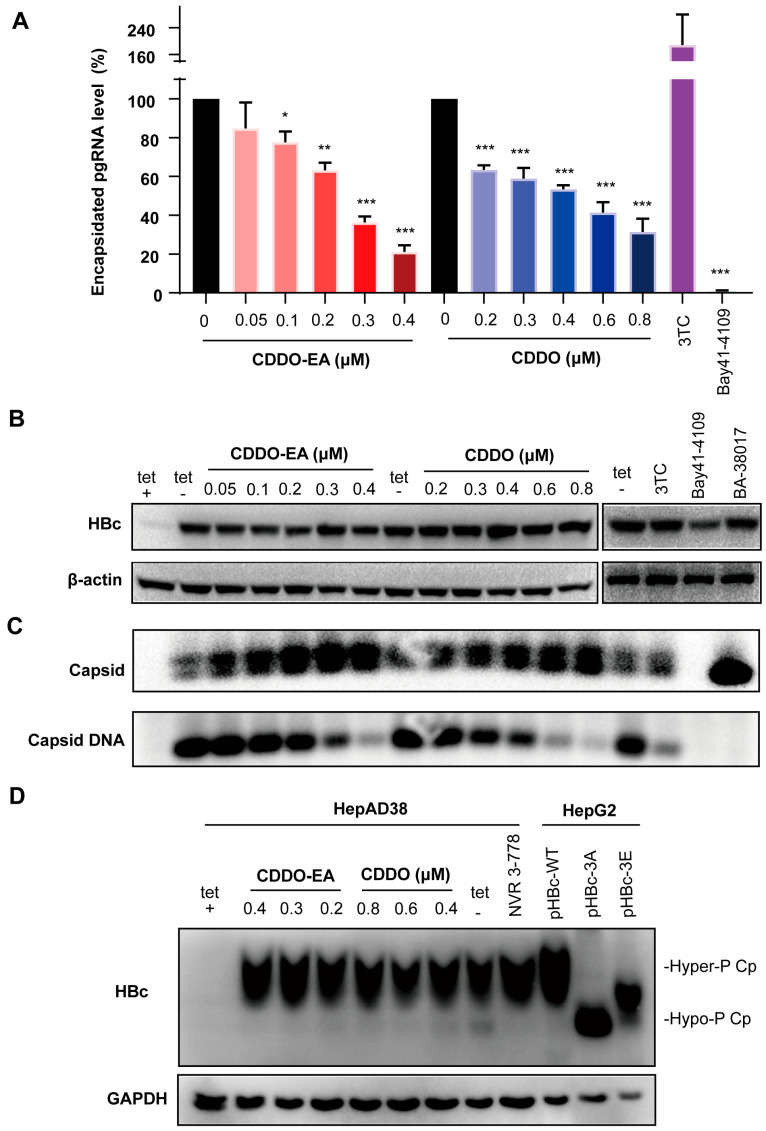
Compounds CDDO-EA and CDDO promote the formation of empty capsids without affecting electrophoretic mobility, and modulate the phosphorylation status of the HBV core protein. HepAD38 cells were cultured in the presence of tetracycline (tet +) or in the absence of tetracycline and mock-treated (0 or tet −) or treated with the indicated concentrations of the compounds CDDO-EA and CDDO for 6 days. (**A**) The encapsidated RNA was extracted and quantified by a qRT-PCR assay. Data are shown as mean ± SD from two independent experiments. * *p* < 0.05; ** *p* < 0.01; *** *p* < 0.001. (**B**) HBV core protein (HBc) expression was detected by Western blotting with a rabbit polyclonal antibody. β-actin served as a loading control. (**C**) The capsids were separated on a 1.8% agarose gel electrophoresis, transferred onto a Hybond-N^+^ membrane and detected by a rabbit polyclonal antibody against HBV core protein. Capsid-associated HBV DNA was detected by using a DIG-labeled probe upon alkaline treatment of the membrane following the particle gel assay. (**D**) Phos-tag gel electrophoresis analysis of HBV core protein phosphorylation status. Total lysates of HepAD38 cells treated with the indicated compounds and the lysates of pCI-HBc-WT, pCI-HBc-3A and pCI-HBc-3E transfected HepG2 cells were resolved by a 12% Phos-tag gel electrophoresis and proteins were transferred onto a PVDF membrane. HBV core protein was detected with a rabbit polyclonal antibody. GAPDH served as a loading control.

Since the HBV pgRNA-containing virus particles in the supernatant come from encapsidated particles within hepatocytes [16], the results thus imply that CDDO-EA and CDDO likely suppress the levels of intracellular encapsidated pgRNA. Indeed, the amounts of cytoplasmic encapsidated pgRNA were dramatically reduced upon CDDO-EA and CDDO treatment (Figure 3A).

To investigate whether the reduction in HBV pgRNA encapsidation is caused by the destruction of nucleocapsid assembly, total viral core protein and capsids in cells treated with selected compounds were next examined. The 3TC treatment did not significantly alter the levels of HBV core protein and capsids but increased the levels of encapsidated pgRNA (Figure 3A–C). In contrast, the representative type I CpAMs Bay 41-4109 treatment reduced core protein level and abolished capsid formation and thereby preventing pgRNA encapsidation and DNA synthesis (Figure 3A–C). On the other hand, treatment of BA-38017 [17], the representative type II CpAMs, did not significantly alter the levels of viral core protein but induced the formation of capsids with faster electrophoresis mobility and reduced capsid-associated HBV DNA (Figure 3B,C). These results are consistent with their antiviral mechanism and previous findings [17]. The total level of viral core protein was not changed after the treatment of the two compounds CDDO-EA and CDDO (Figure 3B). Interestingly, the particle gel assay revealed that either CDDO-EA or CDDO treatment increased the amount of capsids without altering the migration mobility, yet caused a concomitant, dose-dependent reduction in capsid-associated HBV DNA (Figure 3C and Figure S3). Collectively, these results suggest that CDDO-EA and CDDO dose-dependently inhibit the pgRNA encapsidation by affecting the formation of capsids, which precludes the viral DNA synthesis. The results also indicate that inhibition of nucleocapsid assembly by the two synthetic triterpenoid compounds is mechanistically distinct from that of the tested CpAMs.

Studies have shown that core proteins are hyperphosphorylated in empty capsids, whereas they are hypophosphorylated in those containing nucleic acid [18,19]. Moreover, disruption of the core protein dimer–dimer interface by CpAMs or mutagenesis inhibits its dephosphorylation during nucleocapsid assembly [18,19]. The effect of CDDO-EA or CDDO treatment on HBV core protein phosphorylation status was therefore assessed by the phos-tag gel electrophoresis and Western blot assay. As previously reported [19], the core protein mutants with alanine (HBc-3A) or glutamic acid (HBc-3E) substitutions at the three major CTD phosphoacceptor sites exhibited the faster electrophoretic mobility (Figure 3D, three lines on the right). NVR 3-778 [20], another type II CpAMs, was also included as a control and its treatment disrupted the core protein dephosphorylation (Figure 3D). Similar to NVR 3-778, CDDO-EA or CDDO treatment also decreased the levels of hypophosphorylated core protein (Figure 3D).

### 2.3. Properties of Capsids Derived from CDDO-EA or CDDO-Treated Cells

**Figure 4:**
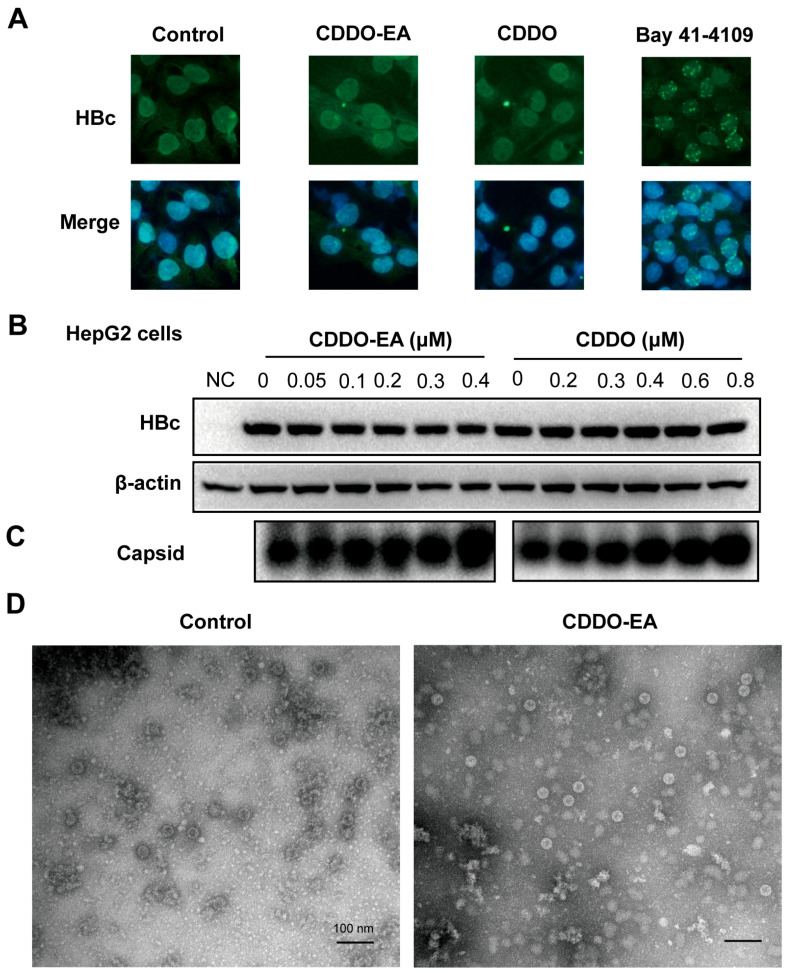
Effects of CDDO and CDDO-EA on HBc localization in HepAD38 Cells, and on HBc expression, capsid assembly, and capsid size in pCMV-HBc-transfected HepG2 Cells. (**A**) HBc expression was detected in HepAD38 cells treated with DMSO (Control), CDDO-EA and CDDO for 6 days by immunofluorescence with a rabbit polyclonal antibody. Compounds 3TC (1 µM) and Bay 41-4109 (2 µM) were used as controls. The fluorescent images were captured using a 40 × objective lens. (**B**–**D**) HepG2 cells were transiently transfected with plasmid pCMV-HBc. Six hours after transfection, the cells were mock-treated or treated with the indicated concentrations of CDDO-EA and CDDO for 72 h. Intracellular core protein (**B**) and capsids (**C**) were detected by Western blotting and a particle gel assay, respectively, with a rabbit polyclonal antibody. β-actin served as a loading control. (**D**) HBV capsids in the cell lysates were purified by sucrose gradient centrifugation and detected by electron micrographs (EM) after negatively stained with phosphotungstic acid. Scale bar indicates 100 nm.

Given that CDDO-EA and CDDO share both similarities and differences with the tested CpAMs, we subsequently examined changes in the localization of HBc protein within HepAD38 cells. Immunofluorescence staining showed that, compared to the control, Bay 41-4109 treatment induced the formation of nuclear punctate foci of HBc protein (Figure 4A). In contrast, the localization of HBc remained unchanged following CDDO-EA or CDDO treatment (Figure 4A).

To further validate the effect of CDDO-EA or CDDO on HBc and capsids, HepG2 cells were transfected with the pCMV-HBc plasmid to enable HBc expression. Western blot analysis confirmed that treatment with either compound did not significantly affect HBc protein levels (Figure 4B). Moreover, particle gel analysis showed that both CDDO-EA and CDDO significantly increased capsid levels (Figure 4C and Figure S4). This phenomenon was consistently reproduced in pCMV-HBc-transfected AML12 and Vero cells (Figure S5), further confirming the results obtained in HepAD38 cells (Figure 3B,C). Collectively, these data demonstrate that CDDO-EA and CDDO can modulate capsid assembly even in the absence of other viral components.

To further explore the characteristics of the compound’s impact on capsid assembly, we next examined the effects on capsid size and morphology. Cytoplasmic capsids were purified from mock-treated and CDDO-EA-treated HepG2 cells transfected with pCMV-HBc via ultracentrifugation and subsequently analyzed by negative-staining EM. In control group cells, the diameters of the capsids were approximately 30 nm, consistent with our previous observations [21]. The capsids in CDDO-EA-treated cells showed no significant differences in morphology or size compared to those in the control groups (Figure 4D).

### 2.4. CDDO-EA and CDDO Specifically Inhibit the Replication of HBV but Not WHV and DHBV

**Figure 5:**
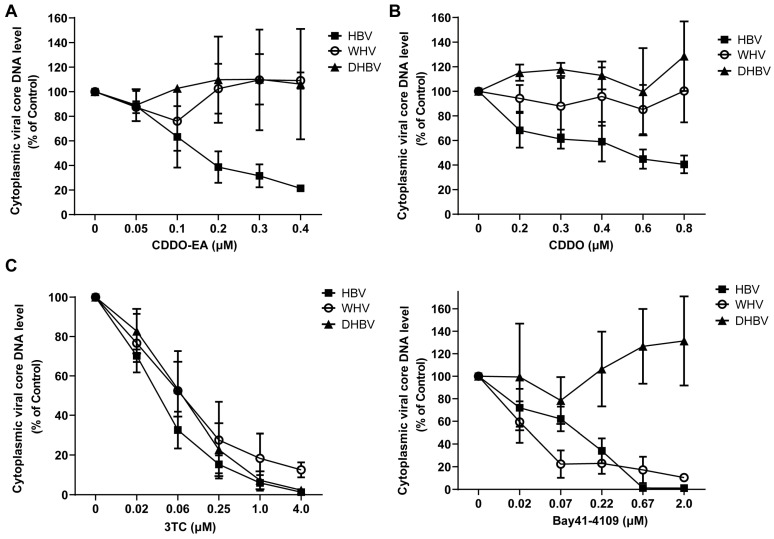
Antiviral spectrum of compounds CDDO-EA and CDDO against hepadnaviruses. HepG2 cells were transfected with plasmids pCMV-HBV, pCMV-WHV and pCMV-DHBV. Six hours post-transfection, the cells were mock-treated or treated with CDDO-EA (**A**), CDDO (**B**), 3TC (**C**) and Bay 41-4109 (**C**) at the indicated concentrations for 3 days. Cytoplasmic viral core DNA was extracted and determined by qPCR assay and expressed as the percentage of the mock-treated controls. The means and standard deviations (*n* = 3–4) were plotted.

The assembly of hepadnaviruses nucleocapsid requires the coordinated interaction among multiple viral and host cellular factors [22,23,24]. To further investigate the mechanism of CDDO-EA and CDDO, the antiviral activity of the compounds against other animal hepadnaviruses (woodchuck hepatitis virus (WHV) and duck hepatitis B virus (DHBV)) were then evaluated in HepG2 cells. This experiment can help to determine whether CDDO-EA and CDDO act on any of the conserved host factors necessary for viral DNA polymerase folding and pgRNA binding. As previously observed [25,26] and consistent with their mechanisms of action, 3TC, a viral DNA polymerase inhibitor, exerted potent inhibition on all three hepadnaviruses, while Bay 41-4109 significantly suppressed HBV and WHV but not DHBV replication. Notably, CDDO-EA and CDDO selectively inhibited HBV replication without significantly affecting WHV or DHBV (Figure 5). Therefore, these results suggest that CDDO-EA and CDDO likely act by directly targeting HBV core protein to modulate nucleocapsid assembly, rather than acting on a conserved host factor.

### 2.5. CDDO-EA and CDDO Alter Capsid Assembly and pgRNA Encapsidation via Specific Interaction with HBV Core Protein

**Figure 6:**
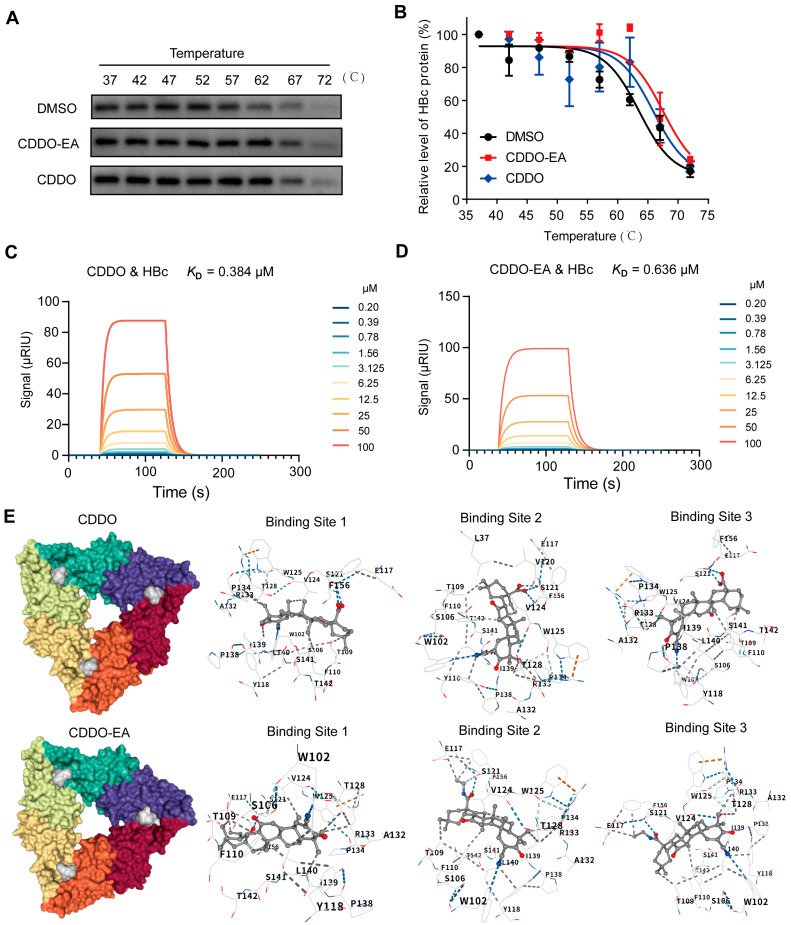
Compounds CDDO-EA and CDDO bind to HBc. (**A**,**B**) Cellular thermal shift assay (CETSA). HepAD38 cell lysate was incubated with CDDO-EA (0.4 μM), CDDO (0.8 μM) or DMSO for 30 min at room temperature. Then the mixtures were heated on a gradient from 37 to 72 °C for 3 min. The supernatant was collected and loaded onto a 12% SDS-PAGE gel. The levels of HBc were detected by Western blot assay (**A**). The quantitative results are shown (**B**). Data are shown as mean ± SD from two independent experiments. (**C**,**D**) The interactions between HBc protein, and the compound CDDO or CDDO-EA were analyzed by a SPR assay. (**E**) Structural simulation and docking analysis. Cartoon mode of interactions between the core protein dimer–dimer interface and CDDO or CDDO-EA are shown.

To confirm the interaction between the CDDO-EA/CDDO and the HBc protein, CETSA and SPR assays were sequentially employed. The CETSA assay showed that, at temperatures above 57 °C, compounds CDDO-EA and CDDO treatments significantly stabilized HBc protein, evidenced by increased expression levels and a right-shifted CETSA melting curve relative to the DMSO control (Figure 6A,B). The results indicated that compound CDDO-EA/CDDO can bind to HBc protein within cells. The SPR assay revealed that compounds CDDO-EA and CDDO exhibited weak binding kinetics to HBc protein (Figure 6C,D), similar to the interaction observed with NVR 3-778 (Figure S6).

The capsid assembly from core protein dimers relies on hydrophobic interactions at the dimer–dimer interface [27,28,29]. In order to comprehend the interaction between CDDO-EA/CDDO and HBc, the molecular docking simulations were conducted. The results revealed that both CDDO and CDDO-EA exhibit high-affinity binding to multiple specific and overlapping sites on HBc, primarily localizing at key dimer–dimer interaction interfaces and intra-protomer structural regions that are critical for capsid assembly and stability (Figure 6E and Table S1). CDDO-EA, with its extended ethyl amide moiety, engages a broader network of residues compared to CDDO, particularly along chain A—a crucial interface involved in interdimeric packing—where it forms interactions with VAL120, SER121, PHE122, VAL124, TRP125, THR128, ALA132, ARG133, and GLU145, suggesting enhanced binding avidity and potential for allosteric disruption. Both compounds target conserved hydrophobic, polar, and aromatic residues that are essential for maintaining the hydrophobic core and hydrogen-bonding networks within and between dimers, including TRP102, THR109, PHE110, ILE139, LEU140, SER141, and THR142. The extensive occupation of these strategic sites implies a multi-mechanistic inhibitory role: steric hindrance at the dimeric interface may directly prevent proper oligomerization, while binding to allosteric sites such as the intra-dimeric hydrophobic pockets and C-terminal domain residues (e.g., LEU154, TYR155, PHE156) could induce conformational shifts that propagate globally and destabilize capsid formation. The more comprehensive interaction profile of CDDO-EA, including its engagement of polar residues like ASN136 and SER141, may facilitate superior binding energy and specificity, potentially translating to more potent disruption of capsid assembly dynamics. These computational insights suggest that both CDDO-EA and CDDO function as molecular wedges that perturb key protein–protein interactions essential for nucleocapsid formation, highlighting their promise as capsid assembly modulators and providing a structural basis for developing novel anti-HBV therapeutics.

### 2.6. Combination of CDDO-EA with 3TC Enhances Their Antiviral Activities

**Figure 7:**
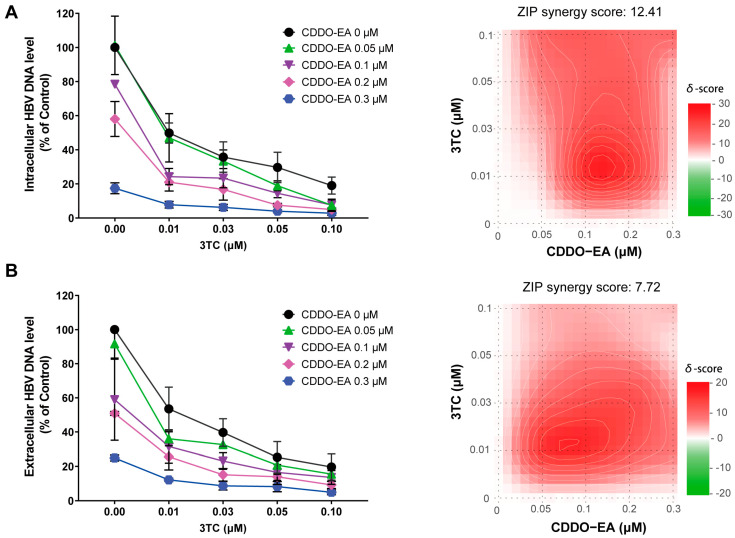
Combination of CDDO-EA with 3TC enhances their antiviral activities. HepAD38 cells were treated with the indicated concentrations of CDDO-EA either alone or in combination with 3TC for 6 days. The level of intracellular (**A**) and extracellular (**B**) HBV DNA levels were quantified by PCR assay and expressed as the percentage of the mock-treated controls. Data are shown as mean ± SD from two independent experiments. ZIP synergy scores were calculated.

Nucleos(t)ide analogues represent the mainstay of therapy and are the current standard of care for chronic hepatitis B. Combination therapy with different mechanisms of action has become increasingly common in current exploratory antiviral treatments for HBV [4,5,7,30]. The combined effects of CDDO-EA with 3TC on intracellular and extracellular HBV DNA were assessed in HepAD38 cells. The qPCR analysis demonstrated a significant synergistic inhibition of both intracellular and extracellular HBV core DNA, with ZIP synergy scores of 12.41 and 7.72, respectively (Figure 7). These results indicate the potential of CDDO-EA as an adjuvant in combination therapy.

## 3. Discussion

The suppression of viral replication with the current antiviral therapeutics results in an improvement of liver diseases and reduction in HCC mortality and morbidity. However, treatment with the current antiviral drugs may ultimately cause the emergence of drug resistance and/or intolerability from long-term, possibly life-long, use of these drugs and rarely achieve the functional cure of chronic HBV infection with HBV surface antigen (HBsAg) seroconversion in the vast majority of patients [2,3,5,7]. Hence, there is a pressing need for novel antiviral therapeutics that are able to induce a durable off-drug suppression of HBV replication or cure HBV infection. To achieve the goals, many great efforts to the discovery and development of therapeutic agents targeting viral components and their interactions with host cellular proteins have been made. For example, targets for inhibition of HBV entry, viral DNA polymerase, or nucleocapsid assembly, degradation of viral mRNA, inactivation of cccDNA function, and induction of HBsAg seroclearance and seroconversion, as well activation of host innate and adaptive immune response against HBV are currently under preclinical or clinical development.

The results of this study provide a novel chemical entity during in the discovery and development of antiviral agents against HBV. We first confirmed the inhibitory effect of the two triterpenoids compounds CDDO-EA and CDDO against HBV by detecting the intracellular and extracellular HBV DNA levels (Figure 1). Because the RT-qPCR assay showed that CDDO-EA and CDDO have no effect on the total cellular HBV pgRNA levels (Figure 2) and did not affect the expression of cellular HBV core protein (Figure 3B), we speculated that these two compounds inhibit HBV DNA replication not mainly through affecting viral pgRNA degradation. However, bardoxolone methyl (CDDO-Me), another synthetic triterpenoid derivative, was reported recently to reduce the levels of intracellular HBV pgRNA and core protein in cell culture [7]. This may be due to differences in experimental systems and further exploration is needed to investigate the exact differences.

HBV capsid proteins are structurally and functionally distinct from all the cellular proteins, thus viral capsid assembly and/or disassembly have been considered to be a new frontier of antiviral targets with highly selectivity [29,30,31,32]. Indeed, several type I and type II CpAMs are currently in clinical trials and demonstrated potent anti-HBV activity. CpAMs have been demonstrated to alter the nucleocapsid assembly and disrupt the encapsidation of pgRNA, thereby affecting multiple steps in HBV replication life cycle. Our further mechanistic study demonstrated that the two compounds CDDO-EA and CDDO suppress HBV replication by significantly inhibiting viral pgRNA encapsidation with a reduction in the DNA-containing capsids but an increase in the empty capsids (Figure 3A,C). Obviously, this phenomenon is different from the characteristics of type I CpAMs such as Bay 41-4109, which can interact with core protein dimers to misdirect the assembly of noncapsid aggregates and in the particle gel assay completely abolish capsid formation. Similar to type II CpAMs, CDDO-EA and CDDO can induce the formation of empty capsids that are devoid of viral genome. It has not been fully known that how type II CpAMs inhibit pgRNA encapsidation and induce the assembly of empty capsids. It has been previously reported that core proteins are hypophosphorylated in pgRNA- and DNA-containing capsids, but hyper-phosphorylated in empty capsids and inhibition of pgRNA encapsidation by sulfamoylbenzaminde derivatives (SBAs) might be due to disruption of core protein dephosphoryaltion during nucleocapsid assembly [18,19]. We demonstrated that CDDO-EA and CDDO also affect the phosphoryaltion status of core protein (Figure 3D).

We and others observed that HBV capsids can be resolved into two species with distinct migration mobility by increasing agarose concentration to 1.5 or 1.8% in particle gel assays and DNA-containing capsids co-migrate with the slower migrating capsids [17,21]. However, unlike other type II CpAMs examined thus far which induced the formation of capsids that migrate either faster or slower in native agarose gel electrophoresis than do wild-type capsids, CDDO-EA and CDDO treatments promoted the assembly of both these two types of capsids but not change their ratio (Figure 3C). Although Type I and II CpAMs disrupt capsid assembly by binding to the same HAP pocket at a dimer–dimer interface, they induce diverse allosteric effect on capsid structure. Type II CpAMs binding leaded to significant tertiary structural changes, while Type I CpAMs binding induced a much more dramatic quaternary rearrangement [27,28,29]. CETSA and SPR assays demonstrated the direct binding of compounds CDDO-EA and CDDO to the HBc protein (Figure 6A,B). Further molecular docking analysis revealed that these two compounds target core protein at key dimer–dimer interaction interfaces and intra-protomer structural regions that are critical for capsid assembly and stability. Collectively, our results establish that CDDO-EA and CDDO exhibit a unique interaction mode with the HBV core protein, which ultimately perturbs viral capsid assembly. However, the specific site critical for CDDO-EA and CDDO binding requires further verification through subsequent amino acid mutation experiments.

Because CDDO-EA and CDDO are Nrf2 activators, the relationship between the Nrf2 activation and inhibition of HBV DNA replication by the compounds should been explored. However, whereas both CDDO-EA and CDDO selectively suppressed HBV replication, they did not affect the replication of either WHV or DHBV in the same cell line. Thus, we speculate that CDDO-EA and CDDO inhibited HBV replication independent of Nrf2 activation in cells. Of course, this speculation requires more experimental evidence to substantiate.

In conclusion, our work reported here suggests that synthetic triterpenoids CDDO-EA and CDDO were identified as a novel antiviral agents against HBV. In addition, these two synthetic triterpenoids inhibit viral pgRNA encapsidation by altering the capsid assembly which is distinct mechanisms from other CpAMs. Obviously, it is necessary to test the antiviral efficacy of CDDO-EA and CDDO in vivo (e.g., HBV hydrodynamic injection or AAV-HBV transduced mouse models) in the future study.

## 4. Materials and Methods

### 4.1. Cell Culture and Reagents

Human hepatoblastoma cell line HepG2 were acquired from the Cell Bank of the Chinese Academy of Sciences (the catalog number: SCSP-510) and maintained in MEM medium (Invitrogen, Carlsbad, CA, USA) containing 10% fetal bovine serum (Gibco, Waltham, MA, USA), 100 U/mL penicillin and 100 µg/mL streptomycin. The HepAD38 cell line, a HepG2-derived stable line capable of tetracycline-regulated HBV genome replication, was grown in the same base medium with additional supplements: 1 µg/mL tetracycline and 400 µg/mL G-418. Induction of HBV pgRNA transcription and DNA replication was achieved by withdrawing tetracycline from the culture medium. Lamivudine (3TC), Bay 41-4109, CDDO-EA and CDDO were procured from MedChemExpress (MCE, Monmouth Junction, NJ, USA). BA-38017 is a gift from Dr. Ju-Tao Guo at Baruch S. Blumberg Institute and has been described previously [17]. NVR 3-778 was synthesized internally.

### 4.2. Plasmids

Plasmids including pCMV-HBV, pCI-HBc-WT, pCI-HBc-3A, pCI-HBc-3E, pCMV-HBc, pCMV-WHV and pCMV-DHBV are generously gifted by Dr. Ju-Tao Guo at Baruch S. Blumberg Institute and have been described in prior studies [17,25].

### 4.3. Cytotoxicity

Cell viability was assessed to determine compound cytotoxicity. Briefly, cells were plated into 96-well plates in complete MEM medium. After 24 h, cells were either left untreated or exposed to a serial dilution of testing compounds, ranging from 4 μM to 0.05 μM, for 6 days. The cell viability was evaluated using the PrestoBlue cell viability reagent, following the manufacturer’s protocol (Invitrogen).

### 4.4. Transient Transfection

HepG2 cells were seeded into 24-well plates and allowed to reach approximately 70% confluence. Transfection was performed using 1.25 µL of Lipofectamine 3000 (Invitrogen) per well to deliver 0.25 µg of the target plasmid(s). Following a 6-h incubation, the medium was refreshed with fresh media or media containing the specified concentrations of test compounds. Cells were then cultured for a further 72 h prior to analysis of intracellular HBV core protein, capsids, and core DNA using the assays described below.

### 4.5. Analyses of HBV Core DNA by Hybridization and qPCR Assays

Cytoplasmic HBV core DNA from HepAD38 cells or transfected HepG2 cells were isolated as described previously [17] and quantified by real-time PCR using TransStart Tip Green qPCR SuperMix (TransGen Biotech, Beijing, China) with forward primer 5′-GGCTTTCGGAAAATTCCTATG-3′ and reverse primer 5′-AGCCCTACGAACCACTGAAC-3′. Amplification was conducted on an ABI 7500 Fast system (Applied Biosystems, Foster City, CA, USA) under these conditions: 94 °C for 30 s, then 40 cycles of 94 °C for 5 s and 60 °C for 30 s. Secreted HBV DNA in the supernatant was extracted and quantified with a commercial qPCR kit (Hunan Shengxiang Biotechnology Co., Ltd., Changsha, China).

For Southern blot analysis [26], DNA samples were separated on a 1.2% agarose gel. After denaturation and neutralization, DNA was transferred to a Hybond-N^+^ membrane (GE Healthcare, Chicago, IL, USA) in 20 × SSC buffer. Membranes were hybridized overnight at 50 °C with a digoxigenin (DIG)-labeled minus-specific full-length HBV probe prepared using the DIG Easy Hyb system (Roche, Basel, Switzerland). Following stringent washes and blocking, the membrane was incubated with an anti-DIG antibody (Cell Signaling Technology, Danvers, MA, USA) and signals were detected using an automated gel imaging system (Bio-Rad, Hercules, CA, USA).

### 4.6. Analyses of HBV pgRNA by RT-qPCR Assays

Total cellular RNA was isolated using TRIzol reagent (Invitrogen). The encapsidated pgRNA was extracted following an established method [33]. The extracellular HBV pgRNA was purified from the supernatant with an EasyPure Viral RNA Kit (TransGen Biotech) according to the kit instructions.

For quantification, RNA was reverse transcribed into cDNA using oligo (dT) primers and GoScript Reverse Transcription Mix (Premega, Madison, Wisconsin, USA). Subsequent qPCR was performed with TransStart Tip Green qPCR SuperMix (TransGen Biotech) under the same amplification condition mentioned above. The β-actin mRNA was used as the internal control for quantifying total cellular viral pgRNA. The HBV pgRNA detection primers were GAGTGTGGATTCGCACTCC (sense) and GAGGCGAGGGAGTTCTTCT (anti-sense). The β-actin mRNA primers were CCAACCGCGAGAAGATGA (sense) and CCAGAGGCGTACAGGGATAG (anti-sense). β-actin mRNA served as an internal control for cellular pgRNA normalization.

### 4.7. Western Blot Assay

Total protein was extracted from cells using 1 × LDS loading buffer (Invitrogen). Cell lysate was resolved by 12% SDS-PAGE and transferred to a polyvinylidene fluoride (PVDF) (0.45 μm, Millipore, Billerica, MA, USA). The membrane was probed with a rabbit polyclonal antibody against N-terminal 144 amino acids (aa 1 to 144) of HBV core protein (GenScrip, Nanjing, China). An antibody against β-actin (Cell Signaling Technology) was used to verify equal loading.

### 4.8. Particle Gel Assay

HBV capsids and associated viral DNA were analyzed by a native agarose gel electrophoresis as reported [34]. Briefly, cells were lysed with buffer containing 10 mM Tris-HCl (pH 7.6), 100 mM NaCl, 1 mM EDTA, and 0.1% NP-40. The lysates were then resolved on non-denaturing 1.5% or 1.8% agarose gels. Capsids were detected by immunoblotting with the anti-HBc antibody (GenScrip, Nanjing, China), while capsid-associated DNA was detected by hybridization with a DIG-labeled, plus-strand-specific full-length HBV probe.

### 4.9. Phos-Tag Gel Assay

To analyze core protein phosphorylation, Phos-tag SDS-PAGE was employed. The 12% Bis-Tris PAGE gel were prepared by incorporating Phos-tag Acrylamide (Fujifilm Wako, Tokyo, Japan, AAL-107, Cat. No. 300-93523) and zinc chloride. Protein samples were electrophoresed in MOPS SDS Running Buffer and transferred to PVDF membrane. After blocking, the membranes were immunoblotted with the anti-HBc antibody (GenScrip, Nanjing, China). GAPDH (Cell Signaling Technology) was detected as loading control.

### 4.10. Electron Microscopic (EM) Analysis of Capsids

For morphological analysis, HBV capsids were purified from cell lysates by sucrose gradient centrifugation as previously detailed [21]. The purified capsids were negatively stained with Uranyless (Electron Microscopy Sciences, Hatfield, PA, USA) and imaged using an Tecnai 12 Spirit/Biotwin electron microscope (LaB6 filament) (FEI, Hillsborough, OR, USA ), operating at 100 kV, with an AMT 2k × 2k CCD camera (FEI).

### 4.11. Cellular Thermal Shift Assay

Cellular thermal shift assay (CETSA) was carried out as previously described [35]. Briefly, protein lysates from HepAD38 cells cultured in complete medium were prepared through freeze–thaw cycles. After centrifugation, cell lysate was incubated with DMSO, CDDO or CDDO-EA for 30 min at room temperature, followed by heating at graded temperatures (37–72 °C) for 3 min. The supernatant was collected and subject to Western blotting to detect remaining HBc levels.

### 4.12. Surface Plasmon Resonance (SPR) Assay

The direct binding interactions between HBc protein, and the compound CDDO or CDDO-EA were measured by SPR on a Reichert4 SPR system (Reichert, Buffalo, NY, USA) using a CM5 chip, following an established procedure [35]. HBc protein was purchased from Sino Biological (Beijing, China)**.**

### 4.13. Molecular Docking

In silico docking studies were performed to model the binding of CDDO and CDDO-EA to HBc. The blind docking process was conducted using the CB-Dock2 web server (https://cadd.labshare.cn/cb-dock2/php/index.php, accessed on 26 December, 2025), an accurate protein–ligand blind docking tool. The three-dimensional structure of HBc was obtained from the Protein Data Bank (PDB ID: 5E0I). The structures of CDDO and CDDO-EA were prepared in SMILES format and converted to 3D conformers for docking. The CB-Dock2 server predicts potential binding pockets and poses using a curvature-based cavity detection method and a hybrid global/local search algorithm. The top 5 binding poses for each ligand were retrieved based on predicted binding energy. The resulting complexes were visualized and analyzed using the built-in interaction analysis module of CB-Dock2, which identifies hydrogen bonds, hydrophobic interactions, salt bridges, and other non-covalent interactions at atomic resolution. The complex with the three lowest binding energy (indicating highest stability) was selected for each ligand for subsequent analysis.

### 4.14. Statistical Analysis

Statistical analyses were performed with GraphPad Prism software (Version 7 and 9). Results are expressed as mean ± SD. Data were analyzed by one-way ANOVA with Holm–Sidak’s multiple comparisons test or unpaired two-tailed Student’s *t*-test (comparisons between two groups). *p* < 0.05 was considered significant.

## Data Availability

The original contributions presented in this study are included in the article/Supplementary Materials. Further inquiries can be directed to the corresponding authors.

## Supplementary Materials

The following supporting information can be downloaded at: https://www.mdpi.com/article/10.3390/ijms27010300/s1.
