# Supplementary material for: Triterpenoids CDDO and CDDO-EA Inhibit the Replication of Hepatitis B Virus by Modulating Nucleocapsid Assembly
Source: Int J Mol Sci. 2025 Dec 27;27(1):300. doi: 10.3390/ijms27010300 (PMC12785804; doi:10.3390/ijms27010300)
Supplement: Supplementary file 1 [file ijms-27-00300-s001.zip › ijms-4029336-supplementary.pdf]

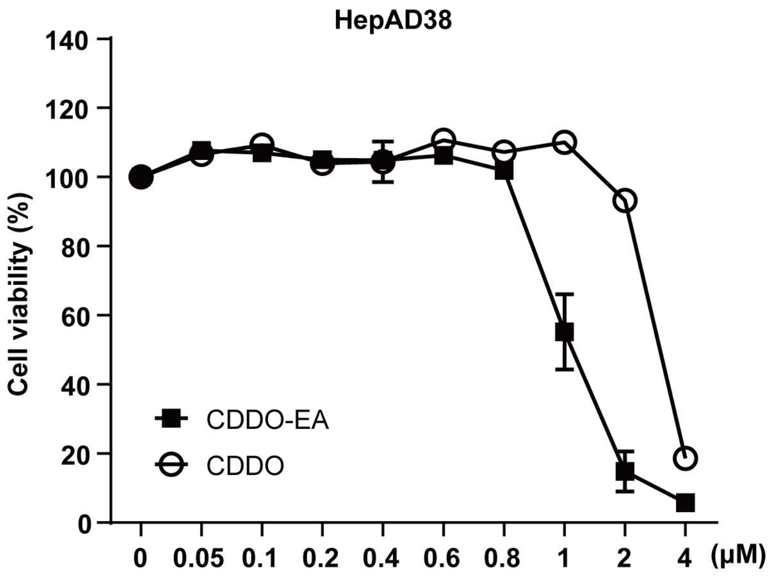

Figure S1. Cytotoxic effect of CDDO and CDDO-EA on HepAD38 cells were determined by a PrestoBlue cell viability reagent.

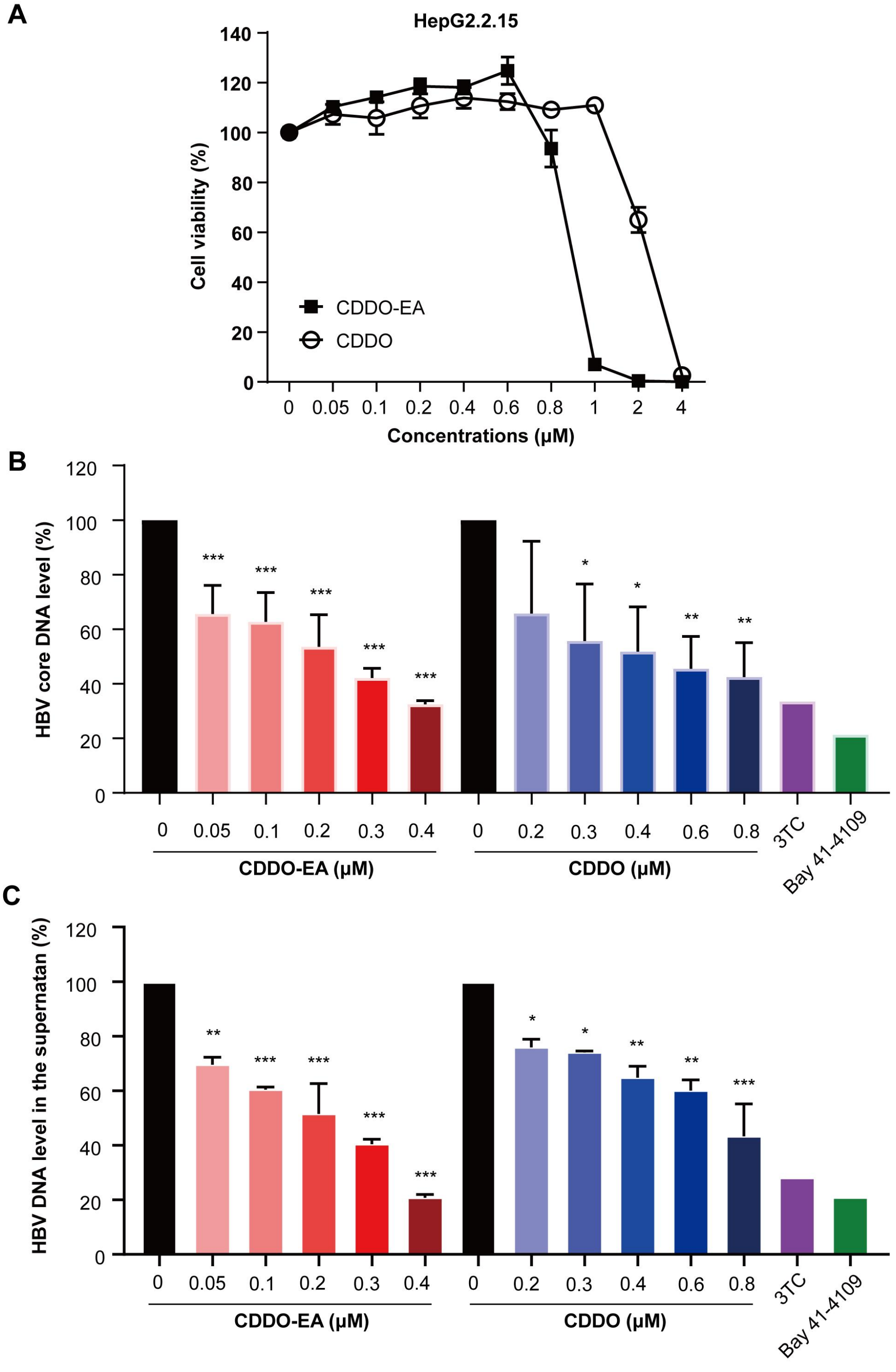

Figure S2. CDDO and CDDO-EA suppress the levels of intracellular and extracellular HBV DNA in HepG2.2.15 cells. HepG2.2.15 cells were treated with the indicated concentrations of compounds CDDO-EA and CDDO for 6 days. **(A)** The cytotoxicity was determined by a PrestoBlue cell viability reagent (n=3). **(B and C)** The intracellular and extracellular HBV DNA were quantified by a qPCR assay and expressed as the percentage of the mock-treated

controls. The means and standard deviations (n=2-3) were plotted. 3TC (1 μM) or Bay 41-4109 (1 μM) was used as a positive control. *P<0.05; **P<0.01; ***P<0.001.

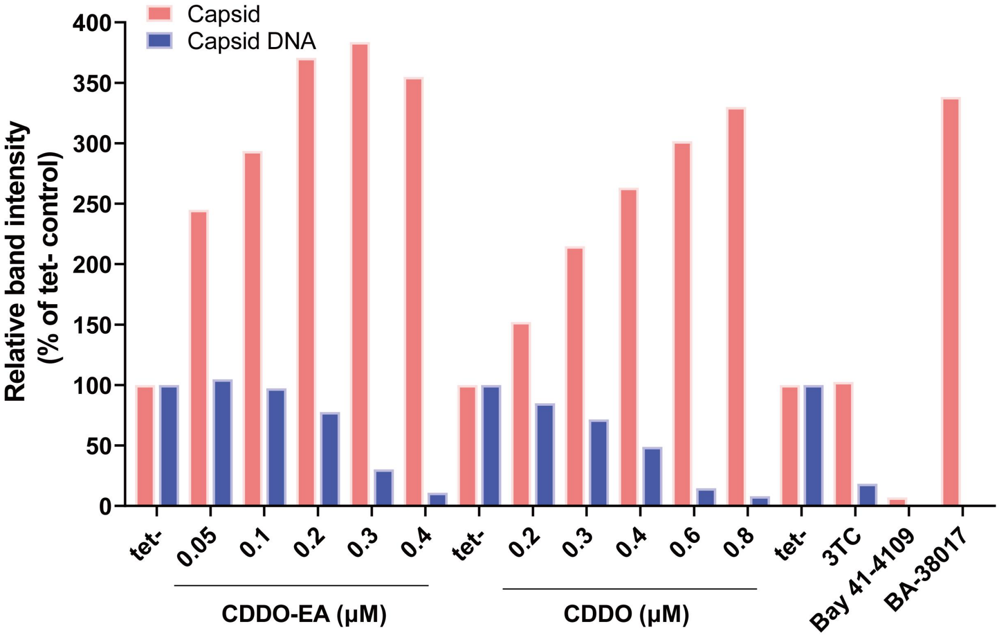

Figure S3. Relative band intensity in Figure 3C.

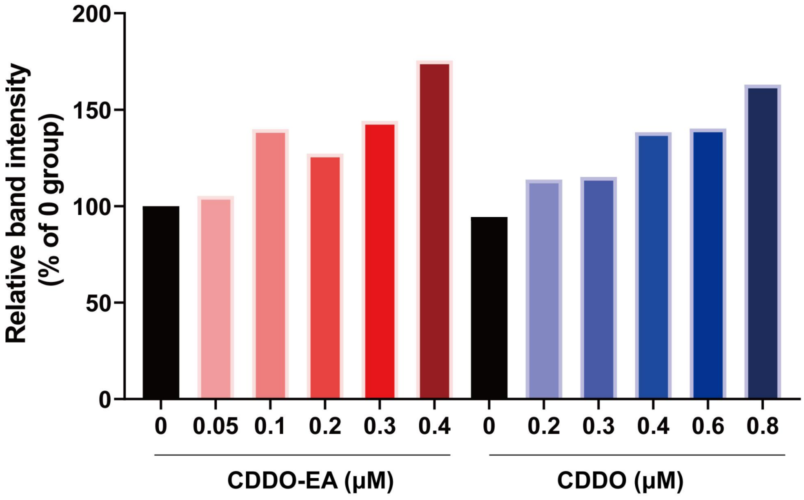

Figure S4. Relative band intensity in Figure 4C.

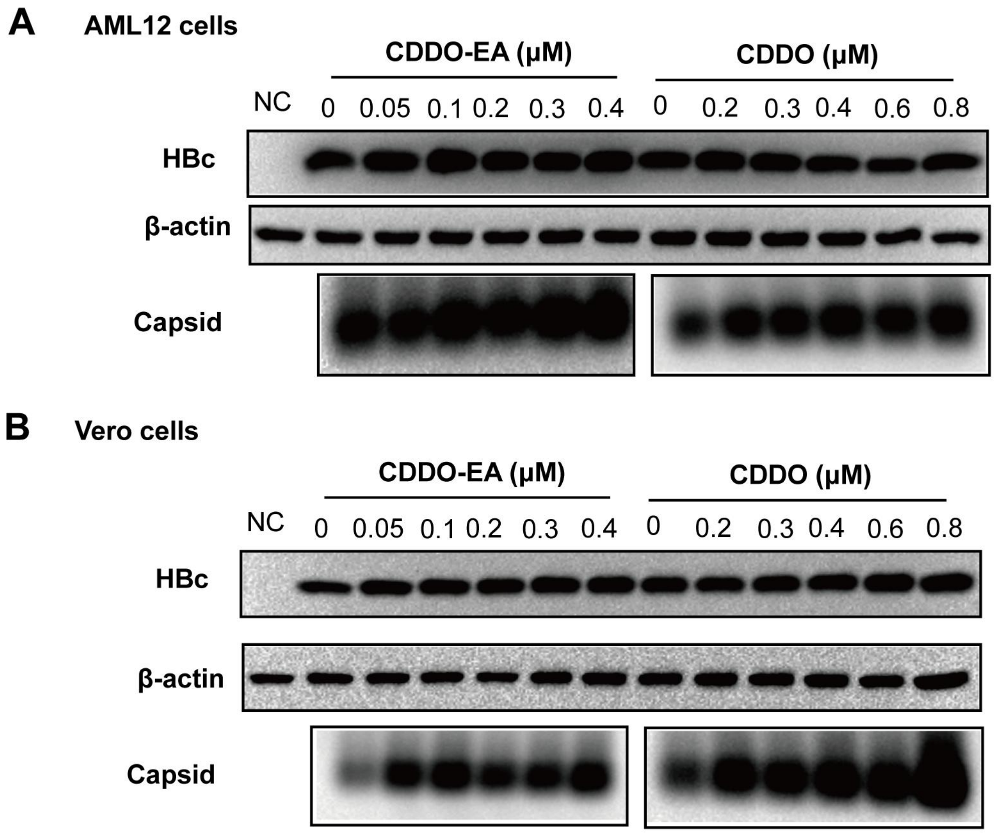

Figure S5. Effect of CDDO and CDDO-EA on the HBc protein and capsids in AML12 cells and Vero cells. AML12 **(A)** and Vero **(B)** cells were transiently transfected with plasmid pCMV-HBc. Six hours after transfection, the cells were mock-treated or treated with the indicated concentrations of CDDO-EA and CDDO for 72 h. Intracellular core protein and capsid were detected by Western blotting and a particle gel assay, respectively, with a rabbit polyclonal antibody. β-actin served as a loading control.

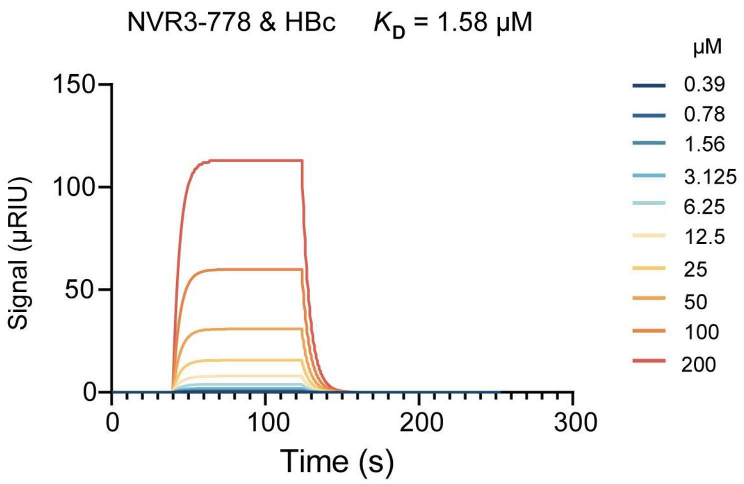

Figure S6. The interactions between HBc protein and the compound NVR3-778 was analyzed by a SPR assay performed on a CM5 chip integrated in the Reichert4 SPR system.

Table S1. Binding sites between the core protein dimer-dimer interface and CDDO or CDDO-EA

Compound	Chain	CDDO	CDDO-EA
Binding site 1	A	GLU:117, VAL:120, SER:121, PHE:122, VAL:124, TRP:125, THR:128, ALA:132, ARG:133, PRO:134, PRO:135, ASN:136, ALA:137, PRO:138, ILE:139, GLU:145, THR:146, THR:147, LEU:154, TYR:155, PHE:156	VAL:120, SER:121, PHE:122, VAL:124, TRP:125, THR:128, ALA:132, ARG:133, PRO:134, PRO:135, ASN:136, ALA:137, PRO:138, ILE:139, GLU:145, THR:146, THR:147, LEU:154, TYR:155, PHE:156
	F	TRP:102, SER:106, THR:109, PHE:110, TYR:118, PRO:138, ILE:139, LEU:140, SER:141, THR:142, LEU:143, PRO:144, GLU:145	THR:33, LEU:37, TYR:38, THR:109, PHE:110, ASN:136, ILE:139, LEU:140, SER:141, THR:142, LEU:143, PRO:144, GLU:145
Binding site 2	B	THR:33, LEU:37, TYR:38, TRP:102, SER:106, THR:109, PHE:110, TYR:118, PRO:138, ILE:139, LEU:140, SER:141, THR:142, LEU:143, PRO:144, GLU:145	PHE:23, PRO:25, ASP:29, LEU:30, THR:33, LEU:37, TYR:38, TRP:102, ILE:105, SER:106, THR:109, PHE:110, TYR:118, PRO:138, ILE:139, LEU:140, SER:141, THR:142, LEU:143, PRO:144, GLU:145
	C	LEU:116, GLU:117, VAL:120, SER:121, VAL:124, TRP:125, THR:128, ALA:132, ARG:133, PRO:134, PRO:135, ASN:136, ALA:137, PRO:138, ILE:139, GLU:145, THR:146, LEU:154, TYR:155, PHE:156	LEU:116, GLU:117, VAL:120, SER:121, PHE:122, VAL:124, TRP:125, ARG:127, THR:128, PRO:129, ALA:132, ARG:133, PRO:134, PRO:135, ASN:136, ALA:137, PRO:138, ILE:139, GLU:145, THR:146, LEU:154, TYR:155, PHE:156
Binding site 3	D	THR:33, LEU:37, TRP:102, SER:106, THR:109, PHE:110, TYR:118, ASN:136, PRO:138,	PHE:23, PRO:25, ASP:29, LEU:30, THR:33, LEU:37, TYR:38, TRP:102, ILE:105, SER:106, THR:109, PHE:110,

		ILE:139, LEU:140, SER:141, THR:142, LEU:143, PRO:144, GLU:145	TYR:118, PRO:138, ILE:139, LEU:140, SER:141, THR:142, LEU:143, PRO:144, GLU:145
	E	GLU:117, VAL:120, SER:121, VAL:124, TRP:125, THR:128, ALA:132, ARG:133, PRO:134, PRO:135, ASN:136, ALA:137, PRO:138, ILE:139, GLU:145, THR:146, THR:147, LEU:154, TYR:155, PHE:156	GLU:117, VAL:120, SER:121, PHE:122, VAL:124, TRP:125, ARG:127, THR:128, PRO:129, ALA:132, ARG:133, PRO:134, PRO:135, ASN:136, ALA:137, PRO:138, ILE:139, GLU:145, THR:146, LEU:154, TYR:155, PHE:156
